# A Review of Planar PIV Systems and Image Processing Tools for Lab-On-Chip Microfluidics

**DOI:** 10.3390/s18093090

**Published:** 2018-09-13

**Authors:** Fahrettin Gökhan Ergin, Bo Beltoft Watz, Nicolai Fog Gade-Nielsen

**Affiliations:** 1 Product Management, Dantec Dynamics A/S, DK-2740 Skovlunde, Denmark; 2 Software Development, Dantec Dynamics A/S, DK-2740 Skovlunde, Denmark; bbm@dantecdynamics.com (B.B.W.); ngn@dantecdynamics.com (N.F.G.-N.)

**Keywords:** micromixers, micro-droplet generation, bio-microfluidics, phase boundary detection, rigid object stabilization, dynamic masking, phase separated µPIV, stereoscopic µPIV

## Abstract

Image-based sensor systems are quite popular in micro-scale flow investigations due to their flexibility and scalability. The aim of this manuscript is to provide an overview of current technical possibilities for Particle Image Velocimetry (PIV) systems and related image processing tools used in microfluidics applications. In general, the PIV systems and related image processing tools can be used in a myriad of applications, including (but not limited to): Mixing of chemicals, droplet formation, drug delivery, cell counting, cell sorting, cell locomotion, object detection, and object tracking. The intention is to provide some application examples to demonstrate the use of image processing solutions to overcome certain challenges encountered in microfluidics. These solutions are often in the form of image pre- and post-processing techniques, and how to use these will be described briefly in order to extract the relevant information from the raw images. In particular, three main application areas are covered: Micro mixing, droplet formation, and flow around microscopic objects. For each application, a flow field investigation is performed using Micro-Particle Image Velocimetry (µPIV). Both two-component (2C) and three-component (3C) µPIV systems are used to generate the reported results, and a brief description of these systems are included. The results include detailed velocity, concentration and interface measurements for micromixers, phase-separated velocity measurements for the micro-droplet generator, and time-resolved (TR) position, velocity and flow fields around swimming objects. Recommendations on, which technique is more suitable in a given situation are also provided.

## 1. Introduction

Microscale total analysis systems or Lab-On-Chip (LOC) devices are closely following the general miniaturization trend in the world. LOC devices have become miniature chemical laboratories that can multiply the number of parallel experiments while reducing the required sample volume. Research and development in LOC devices led to a better understanding of fluid physics in micrometer scales, and, as a result, it is possible to perform many tasks in these small scales with reasonable accuracy [1]. Today, a number of LOC platforms are already commercially available in pharmacology, cancer/stem cell research, genetic engineering, forensics and point-of-care (POC) diagnostics. The LOC platforms often feature complex microfluidic channels and subsystems engineered for a set of specific purposes. These LOC subsystems can perform pumping, mixing, counting, sorting, separation and other similar functions. Two important subsystems for the LOC subsystems are micro-droplet generation systems and micromixers.

The droplet-based microfluidic devices [2,3,4] can be used as micro-reactors in the pharmaceutical industry in the production and testing of new drugs, or perform polymerase chain reaction (PCR) during DNA amplification [5,6,7] or encapsulate [8,9] and transport cancer/stem cells for subsequent counting and sorting [10]. For example, during cell manipulation experiments often the aim is to encapsulate a single cell in a single droplet [11]. Therefore, it is important to disperse droplets with a certain volume and at a certain rate in order to capture one or two cells within each droplet. Additionally, during droplet generation very high velocity and accelerations can be observed at the rupture instance [12], and these can affect the cell integrity. In other cases, droplets are simply used as micro-reactors, where the co-flow acts as a buffer in the chemical reaction [13]. Due to flow physics, local mixing also takes place within the droplet during its formation. In order to characterize these issues, droplet volume estimation, droplet velocity measurements and flow investigations in and around the droplet are often performed using image-based measurement systems [12], in particular using Micro Particle Image Velocimetry (µPIV) systems.

Dedicated micromixer subsystems are almost always found in LOC devices as mixing has a central role in microfluidic processes [14,15,16]. Mixing enhancement is of substantial focus in microfluidics research, as reducing mixing length means smaller LOC devices. Unfortunately, flows tend to stay laminar in micro-scales and mixing is often a result of molecular diffusion between the species. Passive micromixers rely entirely on the fluid pumping energy to induce mixing and therefore the channel geometry is the main design parameter to enhance mixing. Y-type micromixers [17], serpentine (zig-zag shaped) micromixers and omega-shaped micromixers [18] are examples of passive micromixer geometries. On the other hand, active micromixers exploit other forms of energy in addition to the fluid pumping energy: Pressure fields, sound waves, temperature gradients and magneto-hydrodynamic forces. Therefore, active micromixers often provide a better mixing efficiency at the cost of additional components and systems to shorten the mixing length. Among active micromixer alternatives, magnetic micromixers are often preferred, since the mixing energy can be delivered in a non-contact fashion [19,20,21,22]. Once again, µPIV systems are widely used in micromixer research and development.

One other focus area for microfluidics is drug delivery. LOC systems are frequently used in the research and development of new drugs, but there can be challenges in the administration of a new drug to the patient, for example to a cancer patient [23]. The challenge is that often that the drug needs to be administered locally to attack the cancer cells. For this reason, substantial research is focused on bio-microfluidics, micro-robotics and micro-mechanical systems that can be released in the bloodstream to perform targeted drug delivery [24]. In particular, research is focused on the design of magnetotactic biomicrofluidics [23,24,25], biologically-inspired micro propulsion systems [26,27], and programmable self-assembling micro-systems [28]. As a consequence, the importance of research efforts put on the swimming behavior and hydrodynamics of small marine organisms using flagella and appendages [29,30,31] have increased. The flow physics in micro scales has a direct effect on the locomotion efficiency, as the flow around the micro-swimmers are dominated by viscous forces. To put the reader in the right perspective, the following example can be used: the viscous forces experienced by a ~30 µm micro-swimmer in water at 30 µm/s (*Re*~0.001) resembles the forces experienced by an average human swimmer (*h*~1.7 m) with a swim speed of 1 cm/min in molasses [32]. If one wishes to achieve a reasonable swim speed of 1.5 m/s then one has to swim in a substance similar to bitumen [33] with a kinematic viscosity value of 2550 m^2^/s, in order to experience the same forces acting on a micro-swimmer. In other words, it is important to understand how the micro-organisms deal with the viscous forces during locomotion in order to design efficient micro-robots for drug delivery. The swimming mechanics, kinematics and associated flow fields are often investigated using µPIV systems.

From a broad perspective, challenges are encountered during investigations of LOC subsystems or micro-swimmers, and image-based µPIV systems have great potential in addressing these challenges, due to the associated flexibility in image processing. The three main challenges are related with (a) whether the flow is two- or three-dimensional, (b) whether the flow is single-phase or two-phase and (c) the presence of noise or other information types on the image.

The first main challenge is related to whether the flow is two-dimensional (2D) or three-dimensional (3D). Normally flows tend to remain laminar and 2D in micro-scales, and in these cases measuring two-components (2C) of the velocity is sufficient. However, some flows are 3D. For example, well-designed micromixers achieve a short mixing length by producing some 3D vortices in the flow. In such cases, a measurement system that capture all three components (3C) of the velocity field are required. µPIV is an established image-based flow investigation tool that can measure two or three velocity components [34,35,36].

The second main challenge is that often we are faced with a two-phase flow problem (droplet/bubble generation, micro-mixing, micro-swimmers etc.) with a dynamic and potentially complex interface. As demonstrated below in Figure 5b, different flow conditions often exist across the interface where important phenomena take place, and measurement accuracy suffers at the interface, due to the finite size of the measurement volume and crosstalk. In order to get more accurate results, it is necessary to perform measurements in each phase separately. Phase discrimination and phase separation can be achieved using certain image masking strategies. These strategies provide the dynamic interface location in a systematic manner and can be used in object tracking [37,38,39,40].

The third main challenge is the image quality and the presence of multiple layers of information in the acquired images. The strength of image-based measurements lies within the flexibility of the image processing functions. In many single-phase or two-phase flow PIV applications, it is necessary to perform image processing via contrast enhancement [20,21,41,42] or background removal [18,35,43] before performing the (cross-correlation based) PIV analysis. In addition to this, µPIV systems provide the possibility to peel the different layers of information and allow extraction of multiple variables, one at a time, from a single image acquisition. These variables are object position, velocity and accelerations; flow and concentration fields [42,44].

Finally, µPIV systems are quite flexible and scalable—they can be used in three different stages of a product cycle: (a) During the research and development stage to improve the design, (b) during production stage in real-time monitoring (pathogen detection, food monitoring), (c) in the field (point-of-care diagnostics). In the following sections we will present two image-based particle image velocimetry systems and several image processing techniques to address the challenges encountered during various LOC measurement applications.

## 2. Particle Image Velocimetry Systems for Microfluidics

Planar µPIV systems are used to acquire the results presented in this work. The planar (two-dimensional, 2D) µPIV systems are available in two versions; one that measures 2C velocity fields (*U* and *V*), and the other that measures all 3C velocity fields (*U*, *V* and *W*). In the remainder of this paper we will refer to these systems as 2D2CµPIV and 2D3CµPIV respectively. A good review of the planar µPIV systems is provided in Wereley and Meinhart [34], and a brief description of the system configurations used here are provided below.

### 2.1. Two-Component Measurements

**Figure 1:**
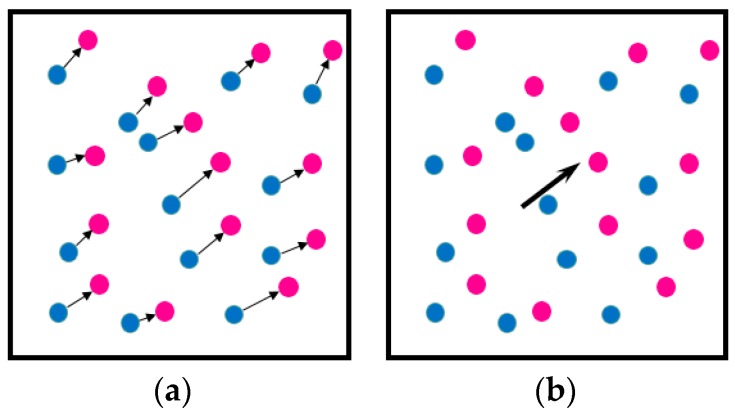
(**a**) Schematic of two-dimensional two-component (2D2C) Particle Tracking Velocimetry (PTV) measurement, where displacement of individual particles are measured (**b**) Schematic of a 2D2C Particle Image Velocimetry (PIV) measurement, where displacement is obtained from the highest peak in the cross-correlation function of a group of particles. Blue dots—initial position; red dots—final position.

The 2D2CµPIV systems use only one camera and the velocity field is estimated using the particle displacements in the object plane:

(1) $$U=\frac{\Delta X}{M\Delta t},\;V=\frac{\Delta Y}{M\Delta t},$$

where *M* is the system magnification, and Δ*X*, Δ*Y* are the pixel displacements in *x* and *y* direction in the image plane and Δ*t* is the time between subsequent images. The displacements in the image plane can be obtained by tracking individual particles (Micro Particle Tracking Velocimetry [45], µPTV), as shown in Figure 1a, or from the highest cross-correlation peak position in the interrogation area (µPIV), as shown in Figure 1b. In this manuscript the discussion will be limited to µPIV only [46,47], which measures the average displacement of all particles in the interrogation area.

**Figure 2:**
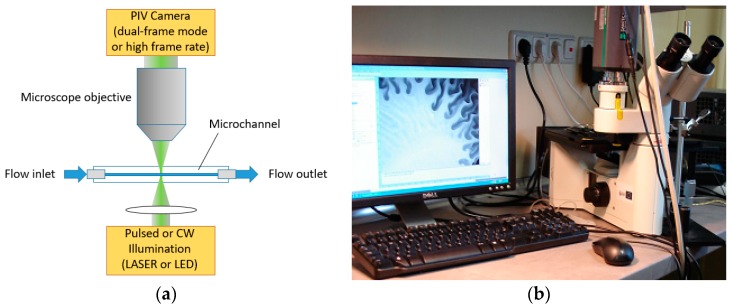
(**a**) 2D2CµPIV measurement in shadow illumination mode. (**b**) A 2D2CµPIV system featuring an inverted fluorescence microscope and a single PIV camera.

The schematic of a 2D2CµPIV measurement system in shadow illumination mode is shown in Figure 2a. Although these systems can be manufactured using an inverted fluorescence microscope and a special filter cube (Figure 2b) [36], most of the results here were obtained using an LED light source in transmission illumination mode. This is often the preferred illumination mode for several reasons: (a) It is possible to get better contrast between the particles and the background using lower light intensities, (b) laser light intensity may disable the micro-organisms in biological applications, and (c) in general LED light sources have lower cost compared to lasers. Because particle shadows are recorded in such setups, it is not mandatory to seed the flow with fluorescent particles. Depending on the application and available hardware, several different illumination/recording combinations are possible:

- A dual-cavity laser or a pulsed LED with a PIV camera running in dual-frame mode,
- A high-repetition rate laser or a pulsed LED with a high-speed PIV camera in single-frame mode,
- A continuous wave (CW) laser or a CW LED with a high-speed PIV camera in single-frame mode. In this last case, the exposure time of the recording is set by the high-speed PIV camera.

Apart from the microscope, illumination and the camera, µPIV systems include a system controller, a synchronization device and software. The 2D2CµPIV system in Figure 2b contains a HiPerformance inverted fluorescence microscope, a HiSense MkII dual-frame PIV camera and a MicroStrobe pulse LED illumination device manufactured by Dantec Dynamics. The microscope is equipped with a safety interlock mechanism, which avoids the laser light propagating to the eyepieces. This particular system was used for the micro-droplet generation experiments [12], the magnetic micromixer experiments [19,20,21,22,42], and the flagellar micro swimmer experiments [40,48].

### 2.2. Three-Component Measurements

**Figure 3:**
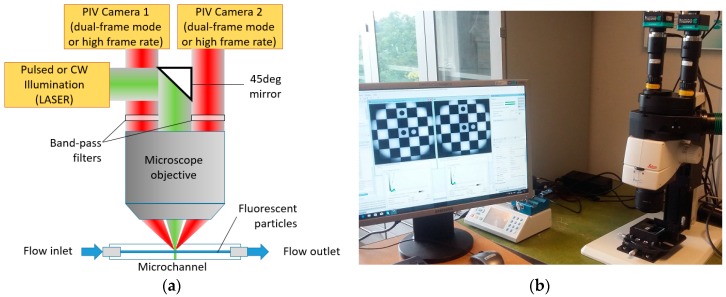
(**a**) Two-dimensional three-component Micro-Particle Image Velocimetry (2D3CµPIV) measurement in back-scatter illumination mode. (**b**) A 2D3CµPIV system featuring a fluorescence stereo microscope, two PIV cameras, a calibration kit, and a syringe pump.

A 2D3CµPIV system [35] uses two cameras (Figure 3) to achieve the stereoscopic viewing principle and therefore is often called Stereo-µPIV system in the literature. The schematic of a 2D3CµPIV measurement system in a fluorescence back-scatter illumination mode is shown in Figure 3a. In this illumination mode, the high light intensity of a laser is often required. The laser light reflects from a 45° mirror, and is focused in the microchannel by the microscope objective (Figure 3a). The flow is seeded with fluorescent particles that are typically coated with a Rhodamine dye that absorbs green wavelengths produced by the second harmonics of the Nd:YAG (λ = 532 nm) or Nd:YLF lasers (λ = 527 nm) and produce a broadband emission in the orange-red spectrum. The fluorescence emission and the Mie scattering are collected by the same objective, and relayed to the two cameras positioned off-center above the microscope objective. Band-pass optical filters are installed in each beam path to block the green Mie scattering from the particles and surfaces, which improves the signal quality. The off-center positioning of the cameras produce a translational stereoscopic imaging configuration [49], where the measurement plane, objective plane and the image plane are parallel. The 3C velocity field for a translational stereoscopic imaging system can be estimated using [49]:

(2) $$U=\frac{\Delta X_{1}\left(x-\frac{S}{2}\right)-\Delta X_{2}\left(x+\frac{S}{2}\right)}{\Delta t\left[MS-\left(\Delta X_{1}-\Delta X_{2}\right)\right]},\;V=\frac{\Delta Y_{1}+\Delta Y_{2}}{2M}\left[\frac{W}{d_{o}}-\frac{1}{\Delta t}\right]-\frac{yW}{d_{o}},\;W=\frac{-d_{o}(\Delta X_{1}-\Delta X_{2})}{\Delta t\left[MS-\left(\Delta X_{1}-\Delta X_{2}\right)\right]},$$

where Δ*X*_1,2_ and Δ*Y*_1,2_ are the displacements in individual camera views, *S* is the distance between beam paths at the lens plane, and *d_o_* is the object distance. In Equation (2) the *U* and *W* components can be calculated purely based on Δ*X*_1,2_ and Δ*Y*_1,2_ information, since the cameras are separated in the *x*-direction. The out-of-plane velocity component, W, can be used to compute *V* accurately. The interframe time (Δ*t*) of the experiment should be small enough to make sure that the out-of-plane particle displacement stays within the measurement thickness. The measurement thickness of volume illuminated µPIV systems (both for 2D2CµPIV and for 2D3CµPIV) are often defined by the correlation depth, *z_corr_*, the thickness where the seeding particles contribute to the correlation function, even though they are out of focus [47]:

(3) $$z_{corr}={\left\{\left(\frac{1-\sqrt{\varepsilon }}{\sqrt{\varepsilon }}\right)\left[\frac{d_{p}^{2}\left[{\left(n/NA\right)}^{2}-1\right]}{4}+\frac{1.49{\left(M+1\right)}^{2}\lambda^{2}{\left[{\left(n/NA\right)}^{2}-1\right]}^{2}}{4M^{2}}\right]\right\}}^{\frac{1}{2}}$$

here, *ε* is the threshold image intensity ratio, i.e., the intensity where the out-of-focus particles begin to contribute to the correlation function divided by the in-focus particle image intensity, *d_p_* is the particle diameter, *n* is the fluid refractive index, and *NA* is the numerical aperture of the objective.

**Figure 4:**
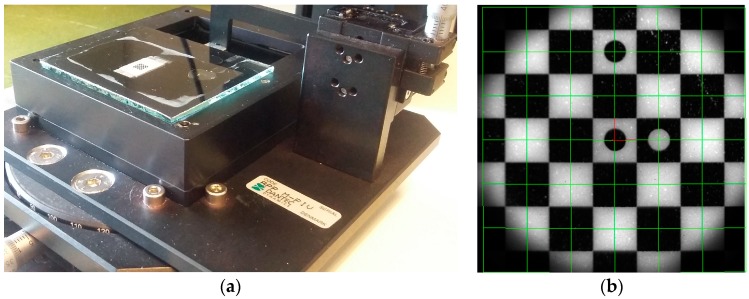
(**a**) Stereoscopic image calibration kit for µPIV. (**b**) Checkerboard calibration target with the assumed coordinate system (red) and metric calibration grid (green). Each tile is 500 µm wide.

The 2D3CµPIV system in Figure 3b was used for the omega micromixer experiment [18], the serpentine micromixer experiment, and the spinning micro-rafts experiment [50]. The 2D3CµPIV system contains an upright fluorescence stereoscopic microscope, a pair of FlowSense EO4MP dual-frame PIV cameras, a DualPower 65-15 dual-cavity PIV laser and a stereoscopic image calibration kit manufactured by Dantec Dynamics. The stereo image calibration kit (Figure 4a) is used for the camera calibration, which is subsequently used for the reconstruction of the displacements in 3D space using the 2D displacements recorded by each camera.

Stereoscopic imaging systems, either translational or rotational, requires the reconstruction of displacements in 3D space from two 2D-displacement observation points (i.e., two cameras). The reconstruction can be a geometric reconstruction or a calibration based reconstruction [49]. The geometric reconstruction relies on the knowledge of the exact geometric placement of the imaging components and uses ray tracing. The calibration-based reconstruction requires the placement of a physical calibration target with a known pattern at least in the center plane of the measurement volume. It is difficult, if not impossible, to place a calibration target in a microchannel and remove it afterwards without damaging the microchannel. Because of this, the pioneering 2D3CµPIV studies relied more on the geometric reconstruction via calibration-correction procedures and ray tracing [35]. The 2D3CµPIV system described here and in References [18,50] does not require any knowledge of the system geometry and uses a dedicated image calibration kit (Figure 4a) for the 3D calibration reconstruction. A checkerboard calibration pattern is etched on the target (Figure 4b) that is placed in a pool of liquid, similar to what is used in the experiments. The basic idea is to replicate the air-glass-liquid interface of the microchannel, to perform the calibration in the surrogate environment and to perform the experiment in the real environment. During stereo image calibration, the checkerboard calibration target is positioned at several distances from the cover-glass using micro-positioners and images are acquired. Carefully placed circular markers allow an unambiguous definition of the planar coordinate axes. The square marker corner detection is performed using the Determinant of Hessian algorithm, which is essentially fitting a second order polynomial to local greyscale variations in an image, where checkerboard crossings are described as saddle points. The Hessian contains 2nd order spatial derivatives of pixel intensities, and local minima of its determinant is used to identify the location of these saddle points. The mapping of the markers in 3D space is achieved by following Soloff et al.’s procedure [51], where *F_x_* and *F_y_* are polynomial mapping functions for each camera view:

(4) $$\left[\begin{array}{c} \begin{array}{c} \Delta X_{1}\\ \Delta Y_{1} \end{array}\\ \begin{array}{c} \Delta X_{2}\\ \Delta Y_{2} \end{array} \end{array}\right]=\left[\begin{array}{ccc} \begin{array}{c} \frac{\partial F_{x}^{1}}{\partial x}\\ \frac{\partial F_{y}^{1}}{\partial x} \end{array} & \begin{array}{c} \frac{\partial F_{x}^{1}}{\partial y}\\ \frac{\partial F_{y}^{1}}{\partial y} \end{array} & \begin{array}{c} \frac{\partial F_{x}^{1}}{\partial z}\\ \frac{\partial F_{y}^{1}}{\partial z} \end{array}\\ \begin{array}{c} \frac{\partial F_{x}^{2}}{\partial x}\\ \frac{\partial F_{y}^{2}}{\partial x} \end{array} & \begin{array}{c} \frac{\partial F_{x}^{2}}{\partial y}\\ \frac{\partial F_{y}^{2}}{\partial y} \end{array} & \begin{array}{c} \frac{\partial F_{x}^{2}}{\partial z}\\ \frac{\partial F_{y}^{2}}{\partial z} \end{array} \end{array}\right]\left[\begin{array}{c} \Delta x\\ \Delta y\\ \Delta z \end{array}\right].$$

Here, four sets of *a_i_* make the coefficients of the polynomial mapping function:

(5) $$\begin{array}{c} F\left(x,y,z\right)=a_{0}+a_{1}x+a_{2}y+a_{3}z+a_{4}x^{2}+a_{5}xy+a_{6}y^{2}+a_{7}xz+a_{8}yz+a_{9}z^{2}+\\ a_{10}x^{3}+a_{11}x^{2}y+a_{12}xy^{2}+a_{13}y^{3}+a_{14}x^{2}z+a_{15}xyz+a_{16}y^{2}z+a_{17}xz^{3}+a_{18}yz^{2}. \end{array}$$

The advantages of using a calibration kit and therefore a calibration-based reconstruction compared to a geometric reconstruction are threefold: (a) The calibration kit allows for a depth calibration, as well as a stereoscopic calibration in each measurement plane, (b) any actual deviations in the assumed values (angles, index of refraction etc.) will be accounted for in the actual image calibration, and (c) it is possible to calculate a reconstruction error, which can be used in the uncertainty estimation.

## 3. Phase-Separated µPIV Measurements

**Figure 5:**
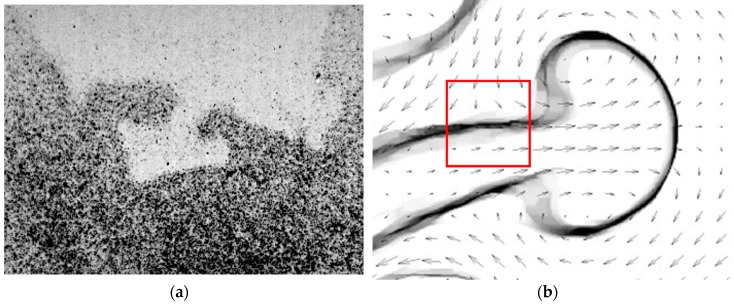
(**a**) Different seeding conditions observed across the reaction zone in a combustion experiment [52]. (**b**) Different flow conditions observed in the mixed-phase PIV results around a mushroom pattern in a magnetic micromixer [22].

2D2CµPIV and 2D3CµPIV systems are quite powerful in obtaining accurate flow field information in single phase flows. However, in two-phase flow investigations, PIV measurement accuracy is often lower along the phase boundaries, i.e., in interrogation windows that contain information from both phases. This is because different seeding density (Figure 5a), velocity magnitude and flow direction (Figure 5b) conditions often exist across the boundary, and the selected correlation peak is either biased towards the wrong phase (crosstalk), or the calculated displacement is erroneously detected as an outlier and is subsequently replaced. Phase separated PIV measurements often minimize this problem, and increase accuracy near the boundary by treating each phase separately. This technique requires for each time step (a) the accurate detection of the phase boundary in consecutive frames, (b) generation of dynamic masks, (c) an accurate PIV evaluation of each phase and (d) recombination of the flow fields.

Dynamic masking [37,38,40,44,48] and phase-separated PIV processing [38,39,53] have become standard routines during two-phase flow field investigations. Different dynamic masking strategies have been reported in the literature, which can be grouped under three categories [54]: (i) During image recording using additional optical systems and components to record one of the phases (optical phase separation, OPS) (ii) during image processing before PIV processing (digital phase separation, DPS), and (iii) post-PIV analysis using differentiators in the cross-correlation function (post-phase separation, PPS). Furthermore, the digital phase separation techniques can be grouped under four categories [38]: (ii-a) Size based discrimination (SBD), (ii-b) histogram thresholding (HT), (ii-c) phase boundary detection (PBD), and (ii-d) rigid object stabilization (ROS) techniques. In challenging cases, dynamic masks are often more successful if both optical and digital phase separation are used. In the following section, examples will be provided only on DPS, and in particular HT-based, PBD-based and ROS-based dynamic masking strategies. For further application examples on dynamic masking in micro-scale and macro-scale two-phase flows please refer to Ergin et al. [38].

## 4. Overview of Application Examples

The results presented here are obtained from seven different experiments the authors collaborated on previously. The applications include micro-droplet generation [12], micromixers [18,21,22,42], microbiological swimmers [44,48,55], and spinning micro-rafts [50]. Multiple quantities were measured for each application, different subsets of the following: 2C and 3C flow fields, concentration fields, interface location, object position, object speed, and object rotation. The experiment setups and measured variables for each case are summarized in Table 1.

Different challenges were observed in each application and a number of tools were applied to overcome these challenges. Low seeding density regions, low Signal-Noise Ratio (SNR) in acquired images, contrast variations, complex morphology changes in two-phase flows, less-than ideal calibration images during stereoscopic image calibration, and Brownian motion are to name a few. Different sets of background subtraction, image enhancement, masking strategies, object/marker detection and object/marker tracking were applied to overcome these challenges. Several statistical and modal analyses were also applied in the post-processing phase, such as Proper Orthogonal Decomposition (POD) and Oscillating Pattern Decomposition (OPD). These are summarized for each case in Table 2.

## 5. Droplet Microfluidics

**Figure 6:**
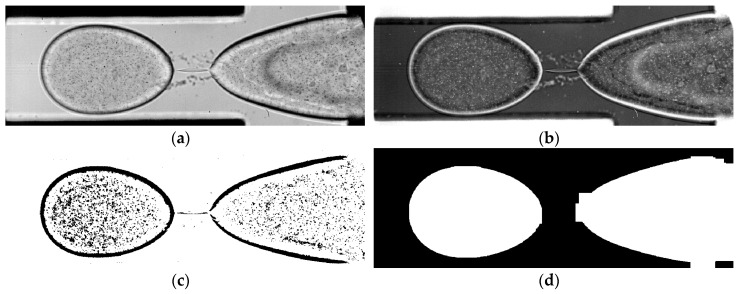
Steps of image pre-processing and dynamic masking: (**a**) Raw image at rupture instance, (**b**) pixel value inversion, (**c**) harmonic mean subtraction and thresholding (inverted), (**d**) dynamic mask. Channel width is 250 µm.

The first example is from a micro-droplet generation experiment [12], where a water and surfactant solution was dispersed in oil (Figure 6a). A movie of this experiment is available in Reference [56]. As can be seen in the movie, the droplet goes through growth, necking, rupture and recoil phases where its shape, size, and morphology changes. Other challenges for this case were, stationary features in the background, non-seeded co-flow, and low SNR. The oil phase is not seeded; therefore, a HT-based dynamic vector masking is applied to reject the spurious vectors in the continuous phase. HT-based dynamic masks are quite popular, because they are relatively simple to apply and are quite powerful in applications where the phase to be masked has a complex shape and undergoing morphological changes (merging of bubbles, rupturing of droplets, etc.). The steps of image pre-processing and dynamic masking are presented in Figure 6 at the rupture instance. The first step is a pixel inversion in order to deal with bright particle images instead of particle shadows (Figure 6b). The second step is to isolate the features in motion (the droplet interface and the seeding particles) from the static background (Figure 6c). This was accomplished by static masking, subtracting the temporal harmonic mean Equation (6), and thresholding:

(6) $$Harmonic\;Mean\left(p_{1},\;p_{2},\ldots ,p_{n}\right)={\left(\frac{1}{n}\sum_{i=1}^{n}\frac{1}{p_{i}}\right)}^{-1}.$$

**Figure 7:**
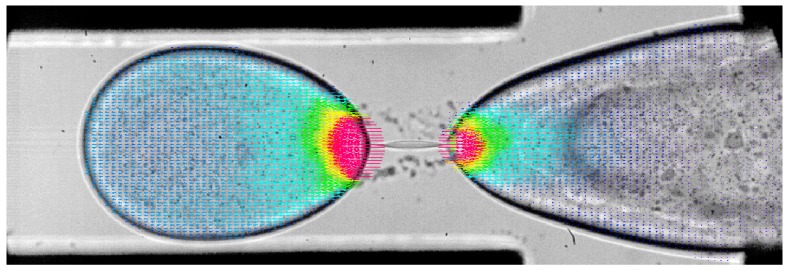
Snapshot of the flow field at rupture during the droplet break-up experiment performed by Carrier et al. [12]. Colors indicate velocity magnitude, max velocity 270 mm/s. Histogram thresholding (HT) dynamic mask is able to follow morphological changes during droplet formation. Channel width is 250 µm.

Harmonic mean subtraction is a more conservative way of reducing background noise, compared to the ordinary arithmetic mean. In order to get the dynamic mask ensemble in Figure 6d, a number of morphology filters (dilations followed by erosions) are applied to fill the gaps between the particles and the interface entirely. The resulting dynamic mask is shown in Figure 6d and the masked vector map is shown in Figure 7. The dynamic mask reported here works quite well for most of the images in the ensemble and can follow the morphological changes, but occasionally fails to cover the dispersed phase precisely for a number of reasons (Figure 6d). This is a typical problem for HT-based dynamic masks, and it will be shown in the next example that PBD-based dynamic masks are more successful in following phase boundaries [53]. Drop tip position, tip velocity, volume, volume change rate was also measured using a dedicated MATLAB script [12]. Further details can be found in Carrier et al. [12].

## 6. Micromixers

### 6.1. Active Micromixers

**Figure 8:**
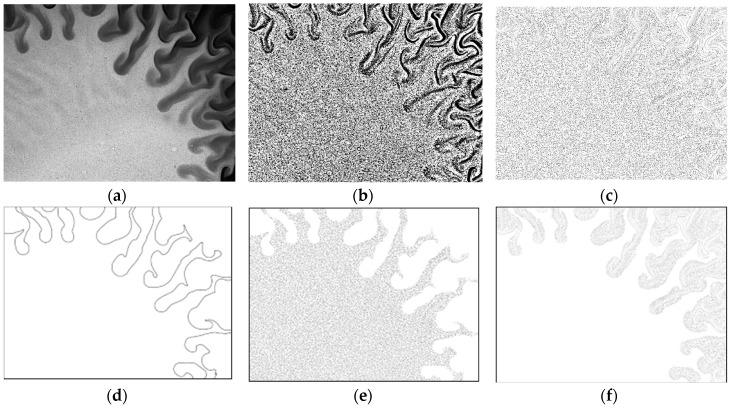
(**a**) Particle image of the magnetic mixing experiment, displaying the magnetic Rayleigh-Taylor instability between the magnetic fluid (dark) and solvent (bright region). (**b**) Processed particle image, after the application of local contrast normalization (LCN). (**c**) Processed particle image after the application of LCN and DoG filter. (**d**) Phase boundary detected by the phase boundary detection (PBD) algorithm, (**e**) water phase, and (**f**) magnetic fluid after phase separation. Field of View (FoV) is 1.6 mm × 1.2 mm.

The second example is from a magnetic micromixer experiment [20,21,22,41,42], where water and magnetic fluid is forced to mix using electromagnetic forces (Figure 8). A movie of this experiment is available in Reference [57]. As can be seen in the movie, a characteristic surface instability (Magnetic Rayleigh-Taylor Instability, MaRTI) takes place where finger and mushroom shaped patterns are observed at the interface. The first challenge is the variable contrast in the raw particle images, i.e., the magnetic phase (darker phase in Figure 8a) contains the same type of seeding particles, but they are not as visible as they are in water phase (brighter phase in Figure 8a). The image contrast can be enhanced using local contrast normalization (LCN, Figure 8b) and Difference of Gaussian (DoG) filter [41]. Briefly, the pixel values are normalized based on the local minimum and maximum pixel values in a 15-pixel neighborhood:

(7) $$\bar{p_{i,j}}=4095\frac{p_{i,j}-p_{min}}{p_{max}-p_{min}},$$

where the pixel values $\bar{p_{i,j}}$ are adjusted to the image depth of a 12-bit image. This operation produces some elongated structures along the phase boundary. This is remedied by a spatial high-pass filter that is essentially the difference between two Gaussian filters, one applied in a 3 × 3 neighborhood and thereafter in a 7 × 7 neighborhood. The integer coefficients of this filter (636× the actual values) are shown in Table 3. The image after LCN and DoG filters is shown in Figure 8c.

The second challenge is related to the flow physics: Different flow conditions exist across the boundary, both in terms of magnitude and direction, and therefore the PIV results are prone to crosstalk in interrogation windows along the phase boundary. For this reason, phase separated PIV analysis is performed in using a PBD-based dynamic masking technique [53] to detect the phase boundary (Figure 8d) and phase separation (Figure 8e,f). PBD-based dynamic masks are more powerful in detecting the in applications where the phase to be masked has a complex shape encountered in surface instabilities, such as those in MaRTI.

**Figure 9:**
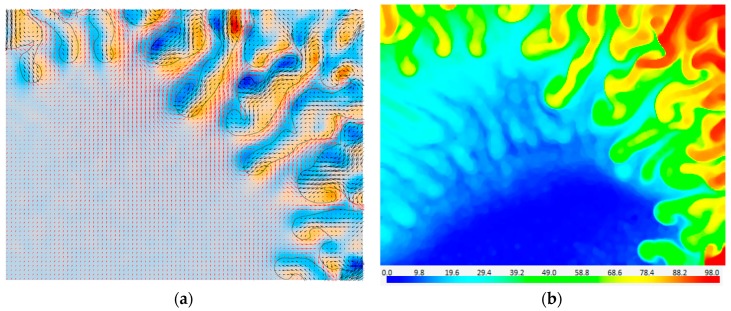
(**a**) Phase-separated PIV measurement results where red vectors are in water, black vectors are in the magnetic fluid (every third vector is shown). Black continuous line is the phase boundary, and the blue and orange colors represent vorticity. (**b**) Magnetic fluid concentration field estimated using absorption imaging. FoV is 1.6 mm × 1.2 mm.

The phase separated PIV results of the magnetic micromixer case is shown in Figure 9a, where red vectors are in water, black vectors are in the magnetic fluid, black continuous line is the phase boundary, and the blue and orange colors represent vorticity. Results of the phase separated PIV evaluation reveal many regions with very sharp velocity gradients and with different flow directions across the boundary, which would not be detected using a conventional mixed-phase PIV evaluation. The phase separated PIV evaluation improves accuracy along the phase boundary.

In PIV measurements the main purpose of the image pre-processing is to isolate the particles from the background (Figure 8c) to get a good SNR for velocity measurements. However, the background image, i.e., bright and dark regions without particles, contains useful information about the concentration of the two species [42]. Concentration information is quite important for mixing problems in the identification of diffusion lengths etc. Just as particle images can be separated from the background, it is also possible to separate the background image from the particles using image-processing functions: Since the seeding particle shadows are recorded with darker gray values, we use a local maximum filter in a 15 × 15 neighborhood. The resulting gray level distribution is a result of absorption of transmitted light by the magnetic fluid. By using the maximum and minimum pixel values in the background image, a region-of-interest (ROI) calibration is performed: The maximum pixel value is observed when the light transmission is at its maximum; i.e., absorption by the magnetic fluid is a minimum. Similarly, the minimum pixel value is observed when the light transmission is at its minimum; i.e., absorption by the magnetic fluid is a maximum. Assuming that the light absorption varies linearly with the magnetic fluid concentration, the entire image is processed to get an estimate on the concentration field during mixing (Figure 9b). In this figure blue color indicates pure water whereas the red color indicates the magnetic fluid.

### 6.2. Passive Micromixers

**Figure 10:**
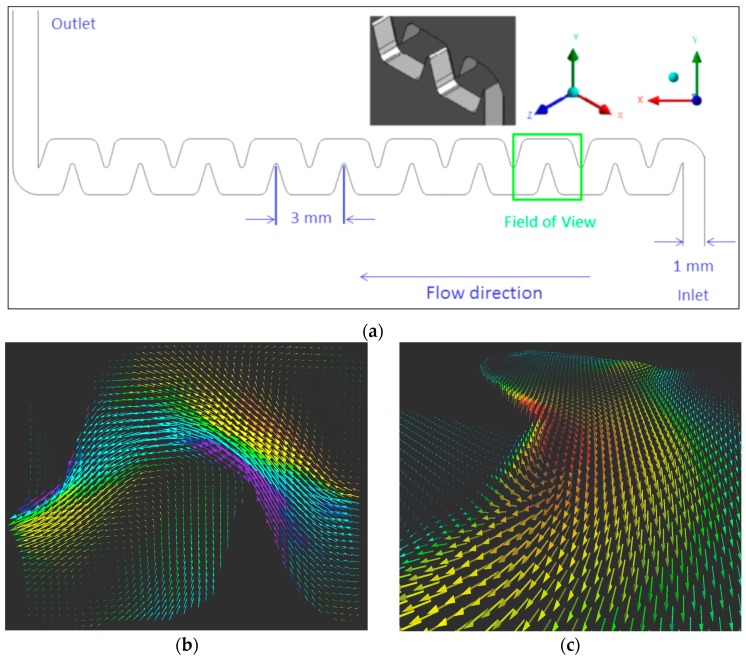
(**a**) Serpentine-micromixer schematic and its dimensions in mm. (**b**) 2D view of the mean velocity field. Average of 910 velocity fields in a FoV of 3.0 mm × 3.0 mm. Colors indicate the out-of-plane velocity component (*w* < ±0.3 m/s). (**c**) 3D view of the mean velocity field (as in Figure 10b) showing a large vortex observed originating from one of the sharp corners of the serpentine micromixer. Colors indicate the magnitude of the velocity vector (0 < *V* < 1 m/s). *Re* = 400.

**Figure 11:**
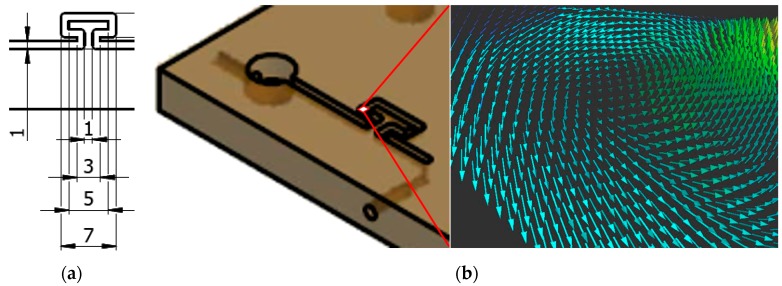
(**a**) Omega-micromixer schematic and dimensions in mm. (**b**) An oblique vortex observed close to one of the sharp corners. Colors indicate the magnitude of the velocity vector.

Mixing is often enhanced in the presence of 3D flow structures, such as vortices. In micro scales the initiation of such 3D microstructures is often suppressed, because of the low Reynolds number inherent to micro scale flows. Passive zig-zag shaped serpentine micromixers (Figure 10a), and omega-shaped micromixers [18] (Figure 11a), are known to induce 3D vortices around sharp corners and thereby enhance mixing efficiency. The aim of this section is to summarize the challenges and proper tools in identification and characterization of such vortices. The next two examples are from passive micromixers where 3C flow field information was obtained using a 2D3CµPIV system. Both passive micromixer experiments are performed at 5× total system magnification, producing a Field of View (FoV) of 3 mm × 3 mm. The results were obtained at the center depth of each microchannel, with slight variations in the experimental setup. These variations are listed in Table 4.

Although both experiments are performed using the same total system magnification, the out-of-plane to in-plane velocity uncertainty ratio [49], and the correlation depth are quite different. The former is due to the choice of the objective working distance. The latter is due to the choice of the seeding particle diameter and the aperture of the optics. The depth of field is adjusted by closing the aperture of the zoom optics of the stereomicroscope. In short, it is quite important to select the right components for the setup already in the design stage of the experiment.

Checkerboard calibration targets were used for the experiments (0.9 mm total size for the omega micro mixer and 4.5 mm total size for the serpentine micromixer). During calibration, a pulsed narrow-band LED device is used for illumination, which proves useful in obtaining sharp calibration images with good contrast. A calibration refinement was performed for both experiments, after which the average re-projection error for left and right cameras were found as less than one pixel. Contrast of raw particle images was enhanced by performing a background subtraction using minimum pixel value found in the ensemble. An ensemble-based static masking technique is also applied [58]. Planar 3C velocity measurements are computed by combining the 2D2C velocity field information from each camera, and using the refined camera calibration information.

The schematic of a serpentine micromixer is shown in Figure 10a, and the planar average 3C velocity field is shown in Figure 10b,c. When the 3C velocity field is visualized from the top view using a conventional 2D viewer (Figure 10b), which is quite normal for investigating planar data, only two large in-plane vortices are immediately apparent. However, when the same data is visualized using a 3D viewer and observed from a particular angle, oblique tip vortices can also be observed originating from the sharp corners of the serpentine micromixer (Figure 10c). Similarly, where conventional 2D views were not useful in detecting oblique vortices in the omega-micromixer, 3D views of the results indicate that oblique micro-vortex systems are present close to the corners of the omega-micromixer (Figure 11b).

## 7. Microscopic Swimmers

In certain applications, it is necessary to track an object in the flow and compute the flow field around it. Flows around biological and man-made micro-swimmers fall into this category. In biological flows, and in biologically inspired propulsion studies, there are often two aims:

- To characterize the flow field around a biological organism and understand how the swimming technique is adjusted to conserve energy and improve efficiency.
- To track the position of the swimmer and compute the velocity and acceleration time history.

A few challenges are worth mentioning in such measurements: First of all, the object in question is most likely present in the PIV raw images. If we are only interested in the flow field information, the successful removal (masking) of the object from the raw particle images is desired, without removing any useful information from the flow field. The second challenge is about the illumination. Most time-resolved µPIV systems use a powerful, pulsed laser as illumination. This type of illumination can easily disable the organism or alter its normal locomotion behavior. A third challenge is related to repeatability of experiments to obtain reliable results. Performing measurements around biological objects means that there is very little control on the locomotion direction, and recapturing of certain dataset is extremely difficult, if not impossible. This requires very accurate PIV algorithms to calculate vector fields from single image pairs. A fourth challenge is related to the timing of the experiment; it is quite difficult to control when the microorganism will swim through the FoV. Therefore, experiments often require special hardware with ring image buffers with post trigger functionality. In this section we will provide two biological flow examples with different swimming techniques (a flagellum swimmer and a breaststroke swimmer) and one rotating micro-raft pair, which is used in dynamic self-assembled micro-systems.

### 7.1. Flagellar Micro-Swimmer

**Figure 12:**
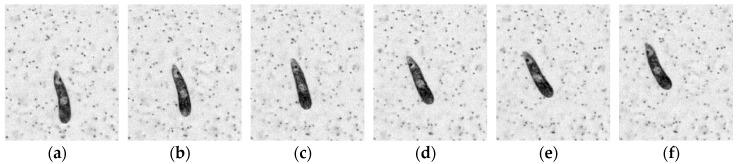
Rolling motion of a 48µm-tall *Euglena gracilis* at various time steps. (**a**) *t* = 2 s (**b**) *t* = 2.16 s (**c**) *t* = 2.32 s (**d**) *t* = 2.48 s (**e**) *t* = 2.64 s (**f**) *t* = 2.8 s.

*Euglena Gracilis* (Figure 12) is known to use a single anterior flagellum (whip-like structure) for swimming, in combination with rolling, stretching and contracting its flexible body [59]. Measurements were taken on a culture of *E. Gracilis* in a 700 µm-deep test channel containing filtered seawater [55]. A lower-power green LED-based pulsed illumination was used in transmission mode (Figure 12a) instead of the high-power pulsed laser illumination, in order not to disable, or affect the normal locomotion behavior of the organism. 1 µm-diameter fluorescent seeding particles were introduced in small quantities until a sufficient seeding density was achieved for PIV. Once again, the seeding density was kept at the lowest possible level in order to avoid a potential change in the normal swimming behavior. A number of individual organisms were present in the full frame, and a region of interest (ROI) is extracted to focus on the swimming technique of a single organism. Three locomotion modes were observed; rolling, stroke and left/right turns.

**Figure 13:**
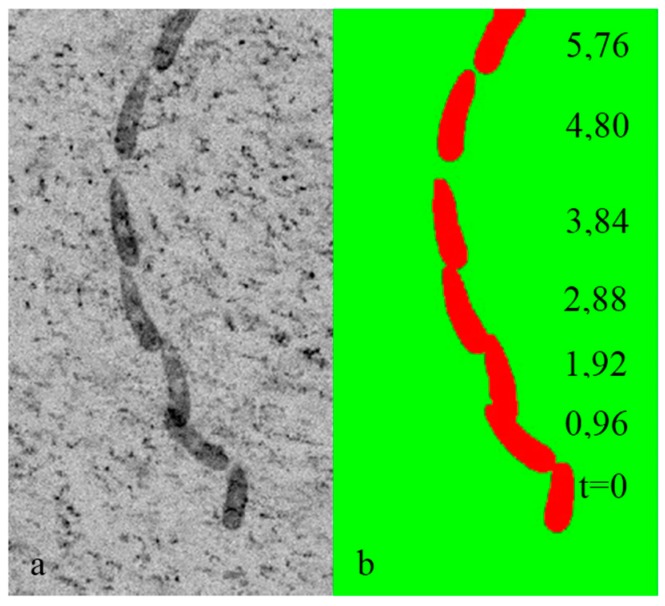
(**a**) Recorded position (**b**) dynamic mask of the 48 µm-tall *E. Gracilis* every 0.96 s.

Figure 12 shows the re-inverted images after an ROI extraction, a pixel value inversion, and background subtraction using temporal minimum pixel values, where the first locomotion mode—rolling motion along the long axis—can be observed. The tracking of the organism however was achieved using a HT-based dynamic mask (Figure 13), where a 48 µm-tall *E. Gracilis* meanders upwards through filtered water; covering a net distance of approximately 256 µm. Details of the dynamic masking procedure can be found in Ergin [37].

**Figure 14:**
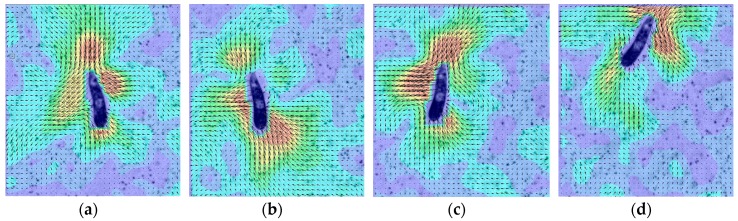
The 48 µm-tall *E. Gracilis* pulling strokes at (**a**) *t* = 1.92 s, (**b**) *t* = 3.52 s, (**c**) *t* = 4.32 s and (**d**) *t* = 5.12 s.

The dynamic mask was primarily generated to obtain more accurate µPIV measurements by separating the object movements and the movement of the seeding particles. For example, when *E. Gracilis* is pulling a stroke using its flagellum, the fluid upstream is pulled towards *E. Gracilis* (downwards), while it is swimming in the other direction (upwards). If not removed, the moving features on *E. Gracilis* image (eyespot, cell nucleus etc.) contributes to the cross-correlation function during the velocity calculation and introduces an error in the flow field. This is well illustrated in Figure 6 in Ergin [37]. Close-ups of the flow field during several stroke instances are shown in Figure 14. In these figures, vectors represent the *U* and *V* components of the flow field and colors represent the magnitude of local velocity, where blue areas represent stagnant flow regions. The stroke is observed first at *t* = 1.92 s, and then at *t* = 3.52 s, 4.32 s and 5.12 s, exactly every 0.8 s or 1.6 s. Every stroke is followed by a rolling motion [55], and the frequency of this corresponds to 1.25 Hz and 0.625 Hz respectively. This is in good agreement with previous studies [55].

The flow field around *E. Gracilis* reveals that the fluid is drawn towards the organism upstream and downstream, and fluid is expelled from the organism on the sides (Figure 14). The upstream flow field is produced by the flagellum pulling a stroke, the main source of propulsion. The downstream flow field can be explained as the wake in the aft of the swimmer. Due to continuity around the organism, the fluid is expelled outwards from the sides. This type of flow field produces four stagnant flow regions, one at each corner of the organism, i.e., due Southwest, Southeast, Northwest and Northeast of the organism.

### 7.2. Breast-Stroke Micro Swimmer

**Figure 15:**
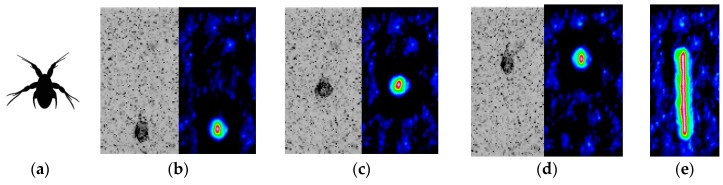
(**a**) Schematic of *Acartia Tonsa* nauplius during a power stroke. (**b**–**d**) Raw particle image and the cross-correlation of the 0.2 mm-tall organism with the (**b**) first, (**c**) middle, and (**d**) last frames of 65 frames. (**e**) *A. Tonsa*’s nearly linear trajectory during the experiment.

*Acartia Tonsa* (Figure 15a) is known to pull double-breaststrokes using the first two pairs of its appendages during the early stage of their life cycle [29]. In an experiment performed at DTU [29,44], a long-distance 2CµPIV system was used to take measurements around a ~220-μm-long individual. A low-power, continuous-wave infrared laser (Oxford Lasers Ltd., Didcot, UK, 808 nm wavelength) was used for illumination. Sheet forming optics was assembled to produce a 150 µm thick light sheet, defining the measurement depth of the experiments. TiO_2_ seeding particles smaller than 2 µm were introduced in small quantities until a sufficient seeding density was achieved. The particle images were recorded on a high-speed CMOS detector (Phantom v210, Vision Research Inc., Wayne, NJ, USA) at a frame rate of 2000 fps and at a resolution of 1280 × 800 pixels. The images were acquired with 11.65× magnification producing a 2.2 mm × 1.4 mm FoV. Since the full frame FoV was only approximately 3 mm^2^, the images were stored in a ring buffer and a manual trigger stopped the acquisition after the organism had passed through the FoV. The first frame of the image ensemble is shown in Figure 15b, where a 0.2 mm tall *Acartia Tonsa* is visible. From this instance, *Acartia Tonsa* propels itself upwards through filtered seawater (Figure 15c,d) by pulling three breaststrokes, covering a distance of approximately 650µm. It is observed that *Acartia Tonsa* moves in an almost-vertical straight line (Figure 15e) and its angular orientation does not change significantly.

The position of the micro-swimmer is recorded by using rigid object tracking and stabilization [38]. The rigid object tracking is based on a cross-correlation routine with sub-pixel accuracy. The basic principle is producing a sub-image that contains the object to be tracked (in this case the *A. Tonsa*’s torso without the appendages), and then correlating this sub-image in the full-frame images of the ensemble. The peak of the cross-correlation function reveals the position of the object with sub-pixel accuracy, and the cross-correlation results for the first, middle and last frames in the ensemble are shown in Figure 15b–d respectively. Integrating the cross-correlation result in time reveals *A. Tonsa*’s nearly linear and nearly vertical trajectory (Figure 15e).

**Figure 16:**
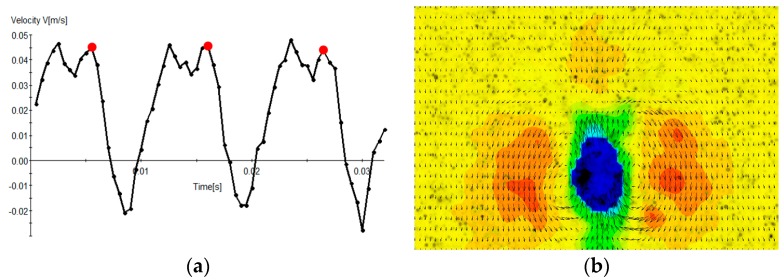
(**a**) *Tonsa*’s swim speed. (**b**) Phase-lock averaged flow field during 2nd power stroke.

The object’s linear speed can be calculated based on the objects position history, and acceleration information can be derived from the speed history (not shown). *A. Tonsa* has a very periodic vertical swim velocity (Figure 16a), and therefore phase-locked averaging techniques can be used during flow field measurements [44]. Once the position of the tracked object is established, a suitable ROI is extracted around the organism, fixing the tracked object in the new frame of reference (rigid object stabilization) [38]. This operation is essentially moving from a global coordinate system to an object-fixed coordinate system. The next step is performing a static masking operation in the object-fixed frame, which is in fact a dynamic masking operation in the global coordinate system. The flow field around the organism is calculated using an Adaptive PIV algorithm throughout the three breaststroke cycles, and phase-locked averaging is performed to get flow field information in a typical swim cycle. There are two velocity maxima and a velocity minimum in a typical swim cycle, corresponding to two power strokes and a recovery stroke (Figure 16a). The phase-locked average flow field results during the second power stroke is shown in Figure 16b. The flow field shown in Figure 16b is the average of velocity maps obtained at three red dots shown in Figure 16a. The flow field around *A. Tonsa* reveal the liquid pulled behind the organism and the downwash of the fluid on both sides as a result of the power stroke.

### 7.3. Magnetically Driven Micro-Rafts

**Figure 17:**
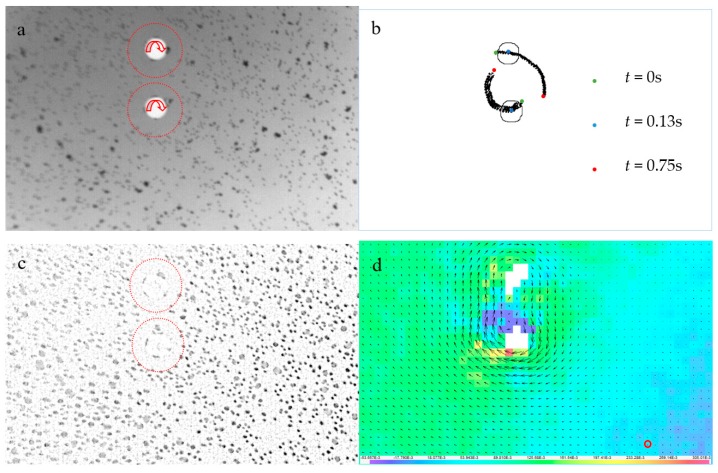
(**a**) Inverted raw particle image of 100 µm-diameter spinning micro-rafts at *t* = 0.13 s. Red arrows indicate rotation direction; red dashed circles show boundary for low seeding concentration. (**b**) The position of micro-rafts during the experiment. (**c**) Processed particle image after dynamic masking and local contrast normalization at *t* = 0.13 s (**d**) 2D3CµPIV results at *t* = 0.13 s, where colors indicate out of plane velocity. Red circle is where the time series is extracted in Figure 18a.

The final application example is related to a pair of spinning micro-rafts from a self-assembling micro-systems study [28]. Time-resolved 2D3CµPIV experiments were performed to measure the free surface motion created, due to a pair of spinning micro-rafts [50]. The 100 µm-diameter cylindrical magnetic rafts were spun in the clockwise direction with a spin rate of 2500 rpm using an external magnetic mixer (Figure 17a). The rafts not only spun on their own axis, but also around each other drawing characteristic circular geometric pattern known as trochoid. One raft moved in a larger radius drawing smaller circles, whereas the other moved in a shorter radius drawing larger circles (Figure 17b). The micro-raft positions were obtained by tracking the center of the dynamic masks using a HT-based masking strategy. The outline of the dynamic mask at *t* = 0.13 ms is shown in Figure 17b. The 3D motion of the free water surface was measured by tracking the floating 5 µm-fluorescent seeding particles. There were differences in the reflected light intensity, since some of the particles coagulated on the free surface. Interestingly, some of the seeding particles coagulated on the rafts’ sidewalls, acting as micro-vanes. In the vicinity of the micro-rafts, large seeding particles were pushed away from the micro-rafts, due to the centrifugal effect and a low seeding concentration region was formed. Local contrast normalization was performed to homogenize the light reflection intensities and recover particle images in the low-seeding density regions (red circles in Figure 17a,c). The stereo image calibration was performed using a 900 µm-wide, square checkerboard calibration target. A calibration refinement process followed to correct for severe camera misalignment between calibration and experiments. Since the rafts were spun in the same rotation direction, a severe shear layer formed between them, where most spurious vectors were computed. These were replaced using a Universal Outlier Detection [60] scheme. The spinning micro-raft pair produced a distinct, 8-shaped vector field rotating around itself in the clockwise direction at a constant speed (Figure 17d).

**Figure 18:**
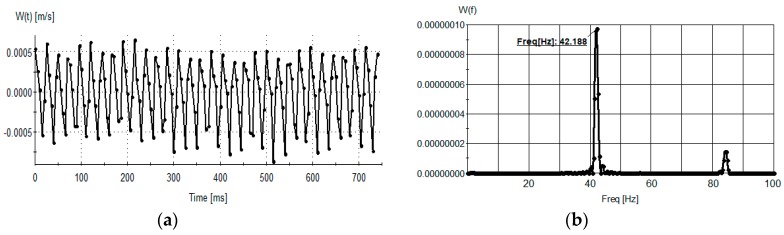
(**a**) Time series of the out of plane velocity component at the red circle in Figure 17d. (**b**) The spectrum of the out-of-plane velocity component in Figure 18a.

The flow disturbances created by the spinning micro-rafts produce a periodic sloshing motion in the far field (Figure 18a). The frequency of the out-of-plane velocity component is measured as ~42 Hz, which is in perfect agreement with the excitation frequency (Figure 18b).

## 8. Discussion, Conclusions, and Recommendations

An overview of current technical challenges and solutions for image-based sensor systems for microfluidics is provided. Both hardware and software side of the solutions have been discussed. Single-, and dual-camera µPIV systems are the current workhorse for the microfluidics flow investigations. High-repetition rate dual-cavity PIV lasers, high-speed dual-frame PIV cameras, single- or stereoscopic-view fluorescence light microscopes, high-resolution synchronizers, dedicated stereoscopic image calibration kits and high-performance system controllers are the current state-of-the-art equipment in many laboratories performing microfluidics research. These systems can provide planar and 3D flow field information in a myriad of applications in LOC devices. These applications include, but are not limited to; mixing, droplet formation, object detection, counting, tracking and flow field measurements in microscopic scales. These applications can serve different purposes in LOC devices and MEMS, such as: Mixing of chemicals, micro-reactors, drug delivery, cell counting, cell sorting, cell locomotion, biologically inspired micro-robotics, and self-assembling micro-systems. In particular, three key applications are covered which are frequently encountered in LOC devices and microfluidics: Micro mixing, droplet formation and flow around microscopic objects. The type of the measurement system was selected based on the application requirements: 2C µPIV systems were used for droplet formation, active micromixer and flow around microscopic objects, whereas 3C µPIV systems were used for passive micromixer and spinning micro-rafts experiments. In general, a 3C µPIV system should be used when 3D flow structures are expected in the flow. The main conclusion is that the image-based sensor systems, in this case PIV systems, are very powerful and flexible in producing results with multiple variables: It was demonstrated that with the current image-based PIV technology, it is possible to obtain the following information in a plane simultaneously:

- The time history of position, velocity and acceleration of swimming objects,
- Up-to 3C, time-resolved and phase separated flow field,
- Concentration field and interface location

Several examples have been provided to demonstrate the image-processing solutions to certain technical challenges encountered in microfluidics. These image processing solutions include: Background removal techniques (by subtracting the temporal minimum, mean, or harmonic mean), contrast enhancement (LCN and DoG filters), dynamic masking strategies (HT-, PBD- and ROS-based) for phase-separated PIV investigations. All these tools improve the accuracy of 2C and 3C µPIV flow investigations in one way or another. For example, background removal improves the signal-to-noise ratio (SNR) of PIV analysis around static features in the FoV. Contrast enhancement techniques should be used in applications where the background has a variable contrast, and improves the SNR mostly in the poor contrast regions. Finally, dynamic masking and phase-separated PIV measurements improve the accuracy along the dynamic phase boundaries. Other post processing tools have also been mentioned, such as phase locked averaging, modal analysis and universal outlier detection in order to remove noise and/or spurious calculations from the results. The reader should refer to the relevant references cited in the bibliography for further details on these pre- and post-processing tools.

## Figures and Tables

**Table 1:** Summary of experiment setups and measured variables.

Experiment	Mag	2C/3C System	Phases	Seeding	Illumination	*f* (Hz)	Measured Variables
Droplet formation	20×	2C	Water + surfactant in oil	1.1 µm latex	Continuous broadband (white), transmission	20,000	Drop tip position & velocity Volume & volume change rate, flow field
Magnetic micromixer	10×	2C	Magnetic fluid in water	1.0 µm Nile-red fluor.	Green pulsed LED, transmission	4	Interface position, concentration, flow field of each phase
Omega micromixer	5×	3C	Water	1.0 µm Nile-red fluor.	PIV laser, back scatter	15	3C flow field statistics, corner vortices
Serpentine micromixer	5×	3C	Water	1–20µm Rhod. B fluor.	PIV laser, back scatter	15	3C flow field statistics, tip vortices, normalized Reynolds stress
Flagellum swimmer	40×	2C	Micro-organism in water	1.0 µm Nile-red fluor.	Green pulsed LED, transmission	12.5	Organism position & speed, rotation rate, flow field during locomotion
Breaststroke swimmer	11.7×	2C	Micro-organism in water	2.0 µm TiO_2_	Infrared CW laser, side scatter	2k	Organism position & speed, flow field during locomotion
Spinning micro-rafts	16.6×	3C	Solid objects in water	5 µm Rhod. B fluor.	TR PIV laser, back scatter	300	Object position, 3C surface displacement field, oscillation frequency

**Table 2:** Summary of challenges and image enhancement tools used during the experiments.

Experiment	Challenges	Image Enhancement and Visualization	Masking Strategy	Edge/Object/Marker Detection	Post-Analysis
Droplet formation	Phase morphology, stationary features in the background, non-seeded co-flow, low SNR	Harmonic mean subtraction, Pixel inversion, grayscale morphology	Dynamic vector masking (HT)	Image ratio threshold	Velocity magnit.
Magnetic micromixer	Variable contrast in two phases, variable pixel intensity in the mixing region	Local contrast normalization, difference of Gaussian	Dynamic image masking (PBD)	Phase boundary detection	Vorticity POD, OPD
Omega micromixer	Image calibration in microscale, marker detection, oblique vortices	Temporal minimum subtraction, 3D viewer	Static image masking	Difference of Hessian	Velocity magnit.
Serpentine micromixer	Image calibration in mm-scale, marker detection, oblique vortices	Temporal minimum subtraction, 3D viewer	Static image masking	Difference of Hessian	Velocity magnit.
Flagellum swimmer	Variable phase shape, Brownian motion	Temporal minimum subtraction, Pixel inversion, median, grayscale morphology	Dynamic image masking (HT)	Histogram threshold	Velocity magnit.
Breaststroke swimmer	Variable phase shape, masking of the appendages	Harmonic mean subtraction	Dynamic image masking (ROS)	Rigid object stabilizat.	Velocity time history
Spinning micro-rafts	Image calibration in microscale, marker detection, low seeding close to objects, particle coagulation, low SNR	Local contrast normalization, median	Dynamic image masking (HT)	Pixel inversion, erosion	Velocity magnit., POD

**Table 3:** Coefficients of the 7 × 7 convolution kernel for the Difference of Gaussian (DoG) filter.

−1	−6	−15	−20	−15	−6	−1
−6	−20	−26	−24	−26	−20	−6
−15	−26	31	84	31	−26	−15
−20	−24	84	176	84	−24	−20
−15	−26	31	84	31	−26	−15
−6	−20	−26	−24	−26	−20	−6
−1	−6	−15	−20	−15	−6	−1

**Table 4:** Settings for the passive micromixer experiments.

Experiment	*Re*	Channel Depth	Objective	Working Distance	Stereo Angle	$\frac{\sigma_{w}}{\sigma }$	Seeding	Cover Thickness	Correlation Depth
Omega micromixer	250	1 mm	2× PlanApo	20 mm	23°	2.4	1 µm	125 µm	99 µm
Serpentine micromixer	400, 800	1.15 mm	1× PlanApo	65 mm	11°	5.0	1–20 µm	2 mm	385 µm
